# Effects of ultrasound-guided erector spinae plane block with dexmedetomidine combined with ropivacaine of the same dose and different concentrations on analgesic effect and rehabilitation quality of patients undergoing thoracoscopic wedge resection of the lung: a prospective, randomized, controlled trial

**DOI:** 10.1186/s12871-022-01768-5

**Published:** 2022-07-16

**Authors:** Chunfang Jian, Yi Shen, Hangxiang Fu, Lizhen Yu

**Affiliations:** Department of Anesthesiology, Longyan First Affiliated Hospital of Fujian Medical University, Jiuyi North Road, Xinluo District, Longyan, 364000 Fujian China

**Keywords:** Dexmedetomidine, Different concentrations, Ropivacaine, Erector spinae plane block, Postoperative analgesia, Rehabilitation

## Abstract

**Objective:**

To investigate the analgesic effect and rehabilitation quality of patients undergoing thoracoscopic wedge resection of the lung under erector spinae plane (ESP) block with dexmedetomidine combined with the same dose and different concentrations of ropivacaine.

**Methods:**

Seventy patients undergoing thoracoscopic wedge resection were randomly divided into groups A (*n* = 35) and B (*n* = 35). To perform ESP block, the groups were administered dexmedetomidine (0.5 μg/kg) combined with 30 mL of 0.33% ropivacaine or 20 mL of 0.5% ropivacaine, respectively, half an hour before general anesthesia induction. We compared the onset time of anesthesia, the block level, and the duration of the block between the two groups. The number of compressions of the analgesic pump within 24 h and 48 h postoperatively and the time of the first compression were noted. The visual analog scale (VAS) scores of static and cough at 0.5 h, 6 h, 12 h, 24 h, and 48 h postoperatively were noted. Furthermore, the 40-item quality of recovery questionnaire (QoR-40) score was recorded at 24 h postoperatively. In addition, we noted the time taken to get out of the bed for the first time, the length of hospital stay, analgesia satisfaction, and the occurrence of related adverse reactions and complications within 48 h postoperatively.

**Results:**

The range of ESP block was wider in Group A than in Group B (*P* < 0.05). Group B had a significantly shorter onset time (*P* < 0.05) and lower static and cough VAS scores at 6 h and 12 h postoperatively (*P* < 0.05); this was associated with significantly fewer compressions of the analgesic pump within 24 h and 48 h postoperatively and significantly more time until the first compression of the analgesic pump was required (*P* < 0.05). Group B was associated with significantly superior QoR-40 scores 24 h postoperatively and significantly shorter time to get out of the bed for the first time than Group A (*P* < 0.05).

**Conclusion:**

Dexmedetomidine combined with 0.5% ropivacaine for ESP block is better than 0.33% ropivacaine for overall analgesia and postoperative rehabilitation of patients undergoing thoracoscopic wedge resection.

**Trial registration:**

ChiCTR2200058114, Date of registration: 30/03/2022.

## Introduction

At present, video-assisted wedge resection of the lung is widely considered an effective method for the treatment of early lung cancer. It offers the advantages of small incision, small trauma, and rapid recovery. However, most patients still report moderate pain, which gets particularly aggravated while coughing activity and affects their postoperative recovery. Erector spinae plane (ESP) block is reportedly safe to use in thoracic surgery; however, the duration of postoperative analgesia with a single local anesthetic is limited. Numerous studies have shown that dexmedetomidine as an adjuvant can significantly prolong the effect of local anesthetics for nerve block, reduce postoperative pain, reduce the dosage of local anesthetics and opioids, and reduce adverse reactions [1–6]. However, few studies have reported on the optimal concentration and dose ratio of dexmedetomidine with the local anesthetic in ESP block [7, 8]. This study was aimed at investigating the effect of dexmedetomidine co-administered with two different concentrations of ropivacaine (amounting to the same dose) for postoperative analgesia in terms of the analgesic effect and rehabilitation quality of patients undergoing thoracoscopic wedge resection of the lung to provide a reference for the concentration and dose of local anesthetics in patients undergoing ESP block for clinical thoracic surgery. The main clinical indicators used in this study were the VAS score at each postoperative time point and the postoperative QoR-40 score. Other indicators included the block level, time of the first compression of the analgesic pump, number of compressions of the analgesic pump, time to get out of the bed for the first time, and length of hospital stay.

## Materials and methods

### General data

This study was approved by the ethics committee of the Longyan First Affiliated Hospital of Fujian Medical University [Approval Number: (2019) Ethical Review Scientific Research No.28], and all patients signed the informed consent form. In addition, this study has been registered in the Chinese Clinical Trial Registry (registration number: ChiCTR2200058114; registration time: 03/30/2022). According to inclusion and exclusion criteria, we selected 70 patients who underwent elective thoracoscopic wedge resection between June 2020 and November 2021. The selection was not influenced by the sex of the patient but was limited to patients aged 30–75 years with a body mass index (BMI) of 18–25 kg/m^2^ and the American Society of Anesthesiologists (ASA) grade of I–III. The exclusion criteria were as follows: patients with block site infection, coagulation dysfunction, history of allergic response to local anesthesia long-term use of anti-inflammatory and analgesic drugs during the recent 3 months, and severe cardiopulmonary, liver, kidney dysfunction and declined to participate.

### Grouping and treatment

In the preliminary experiments, we selected patients undergoing thoracoscopic wedge resection of the lung in whom dexmedetomidine was co-administered with three local anesthetic concentrations of 0.25, 0.33, and 0.5% ropivacaine to induce ESP block before anesthesia; there were five patients in each group. The VAS scores of the three groups at each time point postoperatively were compared. The results showed that the three concentrations were safe and effective, but 0.33% ropivacaine and 0.5% ropivacaine had relatively super analgesic effect. Accordingly, the patients were randomly divided into two groups of 35 individuals. Group A received 0.5-μg/kg dexmedetomidine + 30 mL of 0.33% ropivacaine half an hour before general anesthesia induction; Group B received 0.5-μg/kg dexmedetomidine + 20 mL of 0.5% ropivacaine.

### Anesthesia methods

Patients in both groups were treated with intravenous compound general anesthesia, wherein 0.02–0.03-mg/kg midazolam, 0.2-mg/kg etomidate, 0.2–0.4-μg/kg sufentanil, and 0.15-mg/kg atracurium were injected intravenously. Endotracheal intubation was performed after nitrogen removal and oxygen supply for 5 min. The parameters were set as follows: tidal volume, 8–10 mL/kg; respiratory rate, 10–12 times/min; inspiration and expiration ratio, 1:2; and end-tidal CO_2_ partial pressure, 35–45 mmHg. Furthermore, 5–10 μg of sufentanil was added before the operation. For anesthesia maintenance, intravenous infusion of propofol (50–80 μg.kg^− 1^.min^− 1^) and remifentanil (0.2–0.6 μg.kg^− 1^.min^− 1^) was started. Hemodynamic stability was adjusted accordingly, and end-tidal CO_2_ partial pressure was maintained at 30–40 mmHg; 5 μg of sufentanil was added if necessary. The endotracheal tube was removed once the patient met the extubation indication. Patient-controlled intravenous analgesia was used postoperatively with 2-μg/kg sufentanil + 4-mg/kg flurbiprofen + 10 mg of tropisetron + normal saline diluted to 100 mL; load dose was 3 mL, background dose was 3 mL/h, and additional dose was 3 mL. The locking time was 20 min.

### ESP block

Patients in both groups underwent ESP block half an hour before general anesthesia induction. While the patient was in the lateral position, the high-frequency linear ultrasonic probe (M7 Exp, Shenzhen Mairui Biomedical Electronics Co, Ltd.) was placed in the longitudinal sagittal direction at 3 cm outside the T5 spinous process. The trapezius muscle, rhomboid muscle, erector spinae muscle, and the tip of the T5 transverse process could be clearly visualized from the top to the bottom. Using the in-plane technique, local anesthetics were injected when the tip of the needle reached between the erector spinae muscle and the transverse process. Another anesthesiologist who was blinded to the grouping measured the block plane by acupuncture 30 min after performing the block.

### Outcome measures

The onset time, plane, and duration of the block were recorded. The visual analog scale (VAS) score was used to evaluate the severity of pain. One day before the operation, the anesthesiologist explained the VAS scoring rules to the patients, with 0 representing no pain and 10 representing unbearable pain, and the patients selected a number from 0 to 10 based on their perception of pain. The patients were instructed to press the analgesic device when they perceived their conscious VAS score to be greater than 3 for patient-controlled analgesia. The VAS scores were recorded at rest and while coughing at 0.5 h, 6 h, 12 h, 24 h, and 48 h postoperatively. The number of times the analgesic pump was pressed and the postoperative time point at which the analgesic pump was pressed for the first time were noted at 24 h and 48 h postoperatively. The time taken to get out of the bed for the first time and the time of hospitalization were recorded. The patients were followed up 24 h postoperatively, and at the same time, the QoR-40 questionnaire was administered to score the quality of life. The analgesia satisfaction of patients with postoperative analgesia was recorded on a scale of 0–100 points at 48 h postoperatively. At the same time, the occurrence of adverse reactions or complications, such as respiratory depression, nausea and vomiting, hypotension, bradycardia, and local anesthetic toxicity within 48 h postoperatively were recorded.

### Statistical methods

In our preliminary study, we have proved that 0.33% ropivacaine and 0.5% ropivacaine had relatively super analgesic effect than 0.25% ropivacaine. In the next preliminary experiments, 10 eligible patients with thoracoscopic wedge resection were randomly divided into group A (dexmedetomidine combined with 0.33% ropivacaine, *n* = 5) and group B (dexmedetomidine combined with 0.50% ropivacaine, *n* = 5). The static VAS score at 6 h postoperatively was selected for analysis. The mean static VAS scores at 6 h postoperatively were 1.95 ± 0.80 and 1.25 ± 0.75 in groups A and B, respectively. This score for group B was significantly lower than that for group A(*P* < 0.05). PASS (version 11.0.7, NCSS LLC) for Windows was used to calculate the sample size. The Student *t* test was used, and the group allocation ratio was equal. Then, we calculated that a sample of 24 patients would provide 90% power at a two-sided α level of 0.05. Finally, we recruited 35 patients in each group for a total of 70 patients while taking into consideration the possibility of dropouts and incomplete follow-ups (20%). Therefore, 70 patients were included in this study. SPSS 23.0 (IBM Inc., New York, NY) was the statistical software used for analysis. The measurement data are expressed as mean ± standard deviation ($\overline{\mathrm{x}}\pm \mathrm{s}$). Notably repeated measures analysis of variance (ANOVA) was adopted for between-group comparisons, and multivariate ANOVA was adopted for between-group comparisons at each time point. The counting data were described using rate and ratio, and the chi-square (*χ*^2^) test was used for between-group comparisons. The difference was statistically significant (*P* < 0.05).

## Results

There was no significant difference in age, sex, weight, BMI, operation time, and the ASA grade between the two groups (Table 1). All patients completed the trial. In Group A, the ESP block was performed in the T3–T7 spinal nerve innervation area in 19 cases and in the T2–T8 spinal nerve innervation area in 16 cases. In Group B, the ESP block was performed in the T3–T7 spinal nerve innervation area in 20 cases and in the T2–T8 spinal nerve innervation area in 15 cases. The onset time was shorter for Group B than for Group A (*P* < 0.05; Table 2). There was no significant difference in the ESP block duration between the two groups (Table 2). Compared with Group A, the VAS scores of quiescence and cough at 6 h and 12 h postoperatively were significantly lower in Group B (*P* < 0.05; Table 3), The VAS scores of the two groups did not significantly differ at 0.5 h, 24 h, and 48 h postoperatively (Table 3). the number of times the analgesic pump was pressed as noted at 24 h and 48 h postoperatively was significantly lower in Group B (*P* < 0.05; Table 2), and the time before the analgesic pump was pressed for the first time was significantly longer in Group B (*P* < 0.05; Table 2).

**Table 1:** Comparison of general data between the two groups(*n* = 35)

Groups	Group A	Group B	*p*
Age	54.17 ± 5.89	55.51 ± 5.91	0.896
Gender (male / female)	21/14	23/12	0.621
Weight (kg)	63.60 ± 7.63	61.54 ± 8.20	0.281
BMI (kg/m^2^)	23.49 ± 1.68	23.29 ± 1.62	0.613
ASA (I/II/III)	3/28/4	4/27/4	0.923

Comparison of general data between the two groups

**Table 2:** The level block of erector spinae muscle and the analgesic pump pressure in the two groups (*n* = 35)

Groups	Onset time (min)	Duration (h)	No. of times the analgesic pump was pressed 24 h 48 h	First press time (h)
Group A	14.94 ± 1.28	15.43 ± 2.44	3.83 ± 0.79 5.14 ± 0.97	5.17 ± 1.12
Group B	12.26 ± 1.87 ^a^	16.34 ± 2.42	1.46 ± 0.92^a^ 4.06 ± 0.73 ^a^	5.86 ± 1.00^a^
*P*	< 0.001	0.121	< 0.001 < 0.001	0.009

*Notes*: Compared with group A, ^a^*P* < 0.05

Vertical spinae muscle plane block and analgesic pump compression in the two groups

**Table 3:** Comparison of resting and cough VAS scores and QoR-40 score between the two groups (score, $\overline{\mathrm{x}}\pm \mathrm{s}$ *n* = 35)

Evaluation status	Group	0.5 h	6 h	12 h	24 h	48 h
Static VAS	Group A	0.97 ± 0.62	1.97 ± 0.79	2.63 ± 0.60	2.51 ± 0.51	2.37 ± 0.55
	Group B	0.94 ± 0.60	1.00 ± 0.64^a^	1.86 ± 0.65^a^	2.49 ± 0.66^a^	2.26 ± 0.66
	*P*	0.056	< 0.001	< 0.001	0.196	0.110
Cough VAS	Group A	3.71 ± 0.67	3.86 ± 0.69	4.17 ± 0.75	4.54 ± 0.56	3.97 ± 0.71
	Group B	3.43 ± 0.56	3.29 ± 0.57^a^	3.34 ± 0.59^a^	4.29 ± 0.75	3.66 ± 0.91
	*P*	0.056	< 0.001	< 0.001	0.109	0.110

*VAS* Visual analog score; Compared with group A, ^a^*P* < 0.05

Comparison of resting and cough VAS scores and QoR-40 score between the two groups (score, $\overline{\mathrm{x}}\pm \mathrm{s}$, *n* = 35)

Compared with Group A, the QoR-40 score was significantly higher (*P* < 0.05) and the time taken to get out of the bed for the first time was significantly shorter in Group B (*P* < 0.05); there was no significant between-group difference in the length of hospital stay (Table 4). In addition, ESP block-related adverse reactions and complications did not significantly differ between the two groups (Table 5). In both groups, one patient experienced nausea and vomiting. One patient in Group A had bradycardia and one patient in Group B had hypotension. There were no adverse reactions or complications, such as respiratory depression, infection, nerve injury, and local anesthetic toxicity.

**Table 4:** Comparison of rehabilitation quality between the two groups (*n* = 35)

Group	Group A	Group B	*P*
Time taken to get out of the bed for the first time (days)	1.31 ± 0.13	1.03 ± 0.13 ^a^	< 0.001
Length of postoperative hospital stay (days)	3.04 ± 0.22	2.97 ± 0.20	0.169
QoR-40 scores	170.91 ± 3.90	177.63 ± 4.88 ^a^	< 0.001
Analgesia satisfaction	85.63 ± 2.59	90.00 ± 3.15^a^	< 0.001

Notes: compared with group A, ^a^*P* < 0.05

Comparison of rehabilitation quality between the two groups

**Table 5:** Comparison of adverse reactions and complications between the two groups (*n* = 35)

Group	Group A	Group B	*P*
Nausea and vomiting	1	1	> 0.999
Respiratory depression	0	0	> 0.999
Hypotension	0	1	0.314
Bradycardia	1	0	0.314
Infection	0	0	> 0.999
Nerve injury	0	0	> 0.999
Local anesthetic toxicity	0	0	> 0.999

Comparison of adverse reactions and complications between the two groups

## Discussion

This study adopts a randomized, controlled, and double-blinded method. The results revealed that ultrasound-guided dexmedetomidine combined with different concentrations of ropivacaine for ESP block showed a good analgesic effect in patients undergoing thoracoscopic wedge resection. In terms of the overall postoperative analgesic effect and the rehabilitation quality of patients undergoing thoracoscopic wedge resection of the lung, dexmedetomidine is better combined with 0.5% ropivacaine than with 0.33% ropivacaine for ESP block.

After leaving the intervertebral foramen, each thoracic spinal nerve bifurcates into a dorsal branch and a ventral branch. Among these, the dorsal branch runs behind the transverse costal process and innervates the erector spinae muscle, the rhomboid muscle, and the trapezius muscle upwards. The ventral branch becomes the intercostal nerve. ESP block can completely cover the walking range of the dorsal and ventral branches of the thoracic spinal nerve. Therefore, its block range is wider than that of the intercostal nerve. Notably, 0.2% levobupivacaine and 0.2–0.5% ropivacaine are the commonly used drug concentrations for peripheral nerve block in clinical anesthesia. Relevant studies have used these concentrations of these local anesthetic solutions to observe the analgesic effect after ESP block [9–13]. Compared with levobupivacaine, ropivacaine is characterized by fewer cardiotoxic effects and a probably greater margin of safety [14]. In addition, levobupivacaine is characterized by an outstanding myotoxic potential [14]. The preliminary experiments showed that ESP block with 0.33 and 0.5% ropivacaine had a better analgesic effect than that with 0.25% ropivacaine. Therefore, this study used ropivacaine at 0.33 and 0.5% concentrations, which are commonly used clinically, for ultrasound-guided ESP block and observed the analgesic effect of concentrations after thoracoscopic wedge resection when the overall dose administered was the same. In this study, the block plane was measured 30 min after ESP block because the average blood concentration of ropivacaine peaked 30 min after administration [15].

Dexmedetomidine is a new type of high-selectivity α2 adrenoceptor agonist and has sedative, analgesic, antianxiety, and antisympathetic properties, which are beneficial for the postoperative recovery of patients. The analgesic mechanism of dexmedetomidine mainly involves constricting the surrounding blood vessels to delay the absorption of local anesthetics [16]. In addition, dexmedetomidine directly affects the activity of peripheral neurons by acting on the adrenergic receptor (peripheral apparatus) to enhance the time effect of local anesthesia [17, 18]. Finally, dexmedetomidine maintains the hyperpolarization potential of the cell membrane by inhibiting hyperpolarization-activated cation current to inhibit the return of membrane potential to the resting potential level [19]. Gao et al. [3] showed that dexmedetomidine (1 μg/kg) when added to 0.5% ropivacaine can prolong the ESP block time of patients undergoing thoracoscopy by approximately 120% (approximately 18 h), which is significantly longer than the ESP block times in the group using 0.5% ropivacaine alone and the group with dexamethasone (10 mg) added to 0.5% ropivacaine. In the present study, the concentration of dexmedetomidine was 0.5 μg/kg in Group B, and the ESP block duration was slightly lower than that reported by Gao et al [3] Our preliminary experiments findings and several previous studies [3, 20–22] have shown that dexmedetomidine (1 μg/kg) when added to local anesthetics can significantly prolong the nerve block time, stabilize the intraoperative hemodynamics, significantly reduce the postoperative VAS score, and significantly limit the administration of postoperative analgesic drugs. Vorobeichik et al. found that 50–60-μg dexmedetomidine dose can maximize the duration of sensory block and minimize hemodynamic side effects. No patients had any neurological sequelae, and the evidence of sensory block was of high quality [23]. Abdulatif et al. used dexmedetomidine as an adjuvant at 50 μg and 75 μg doses. The 50-μg dose was associated with lesser time before postoperative morphine was needed, prolonged time to achieve sensory block, and the lesser time before the first rescue analgesia was administered; however, increasing the dose of dexmedetomidine to 75 μg was associated with an increased risk of hypotension [24]. Therefore, when dexmedetomidine at high doses is used as an adjuvant for nerve block, we should be vigilant against the occurrence of adverse reactions, such as hypotension and bradycardia. Therefore, 0.5-μg/kg dexmedetomidine was used in this study.

Ultrasound-guided ESP block is a new fascial space block. It offers the advantages of simple operation, safety, high success rate of block puncture, and few operation-related complications [25]. Fredrickson et al. [26] found that concentration and volume (and thus mass) of local anesthetics were clearly associated with prolonged analgesia. In this study, it was also observed that the effect of 0.5% ropivacaine was significantly faster and lasted significantly longer than that of 0.33% ropivacaine, which is consistent existing literature [27]. However, Hillenn Cruz Eng et al. report that the mass of local anesthetics rather than their concentration or volume is the most important determinant of peripheral nerve block onset and duration [28]. Deng et al. compared three concentrations (0.2, 0.3, and 0.4%) of ropivacaine to improve thoracic nerve block in radical mastectomy. The results showed that the postoperative pain scores associated with 0.3 and 0.4% concentrations were significantly higher than those associated with 0.2% concentration (*P* < 0.05), and there was no significant difference between 0.3 and 0.4% concentrations [29]. In the present study, Group B patients were found to have pressed the analgesic pump significantly fewer times than Group A patients at 24 h and 48 h postoperatively. Furthermore, the time before the patients felt the need to press the analgesic pump for the first time was longer in Group B than in Group A. In addition, the VAS score of Group B patients at 6 h and 12 h postoperatively was lower than that of Group A, indicating that the analgesic effect of 0.5% ropivacaine was better than that of 0.33% ropivacaine for the same overall dose administered, particularly within 12 h from the operation. Appropriate postoperative analgesia is associated with greater comfort, reduced dosages of postoperative analgesics, early resumption of activities, reduced risk of pulmonary complications, and shorter the length of hospital stay [3]. There was no significant difference between the two groups at 24 h and 48 h postoperatively, which may be attributed to the limited duration of a single ESP block. Continuous ESP block can be explored for its benefits and limitations in future research. In this study, there was no significant difference in postoperative ESP-related adverse reactions and complications between the two groups.

QoR-40 is an effective patient-centered multidimensional quality assessment tool [30]. Myles et al. reported that an increase of 6.3 or more in the overall QoR-40 score represents a clinically relevant improvement in the quality of postoperative recovery [31]. In this study, the QoR-40 score of patients in Group B was 6.7 points higher than that of patients in Group A (*P* < 0.05). In Group B, the time taken to get out of the bed for the first time was reduced, and the satisfaction scores of patients with the analgesic effect were higher. These findings were considered to be attributed to the lower VAS score and better analgesia in Group B. In addition, in Group B, the number of times the analgesic pump was pressed postoperatively was fewer and the use of opioids was lesser, which can reduce the adverse effects caused by opioids, can improve the comfort and satisfaction of patients with the analgesic effect, and is conducive to the postoperative rehabilitation of patients, which is consistent with the existing literature [32–34]. However, the two groups did not significantly differ in terms of the length of postoperative hospital stay. This finding could be influenced by the small sample size, and this aspect needs to be further studied in future research.

The inadequacies of this study are limited by the research conditions. In this study, acupuncture was used to determine the block plane, which was relatively subjective; however, the time from the completion of ESP to patients’ postoperative pain is regarded as the duration of ESP block analgesia. In addition, this study was a single-center study with a small sample size, and its conclusion needs to be further confirmed by expanding the sample size. Further large studies are needed to assess the safety and efficacy of the ESP block.

## Conclusions

Dexmedetomidine combined with 0.5% ropivacaine provided effective pain control postoperatively and reduced the need for rescue analgesia. It also more significantly improved the postoperative rehabilitation quality for patients undergoing thoracoscopic wedge resection of the lung.

## Data Availability

The datasets used and analyzed during the current study are available from the corresponding author on reasonable requests. The datasets generated during and/or analyses during the current study are also available in the Research Manager.

## Abbreviations

ESP: Erector spinae plane
VAS: Visual analog scale
QoR-40: The 40-item quality of recovery questionnaire
BMI: Body mass index
ASA: American Society of Anesthesiologists
ANOVA: Repeated measures analysis of variance
